# The polymerization of nitrogen in Li_**2**_N_2_ at high pressures

**DOI:** 10.1038/s41598-018-31355-z

**Published:** 2018-09-03

**Authors:** Jie Zhang, Xianlong Wang, Kaishuai Yang, Ya Cheng, Zhi Zeng

**Affiliations:** 1 Key Laboratory of Materials Physics, Institute of Solid State Physics, Chinese Academy of Sciences, Hefei, 230031 P. R. China; 2 University of Science and Technology of China, Hefei, 230026 China; 3 Beijing Computational Science Research Center, Beijing, 100084 China

## Abstract

The polymerization of nitrogen can be used as high energy density materials. The crystal structures of Li_2_N_2_ at high pressures are explored by using the first-principles method combined with evolutionary algorithm. The phase transitions Pmmm → Immm → Pnma → Cmcm-1 → I4_1_/acd are predicted in the pressure range of 0–300 GPa. Enthalpy calculations reveal that the tetragonal phase I4_1_/acd containing the spiral nitrogen chains is stable above 242 GPa, indicating that the polymerization of nitrogen is realized in Li_2_N_2_ under pressure.

## Introduction

Nitrogen-related compounds attract great interesting for its potential application in high energy density materials (HEDM). The bond energies of the N-N single bond (160 kJ/mol) and double bond (418 kJ/mol) are much less than that of the triple bond (954 kJ/mol) of N_2_. The polymeric nitrogen, in which nitrogen atoms form polymer chains and three-dimensional networks, contains the N-N single and N=N double bonds. Therefore, huge energy will be released when the polymeric nitrogen transformations to N_2_ with the triple bond without producing pollutants. For decades, the polymeric nitrogen was an interesting subject for both experimental and theoretical researchers^1–6^. The monatomic nitrogen was firstly predicted by McMahan *et al*.^1^. The cubic gauche structure (cg-N) with N-N single bonds was successfully synthesized above 110 GPa and 2000 K^2^, which prompts the further study on nitrogen-related materials. Recently, alkali metal-nitrogen systems are extensively studied and new compositions are reported theoretically^7–11^.

As the lightest element in alkali metal, lithium can form different nitrides with nitrogen including Li_3_N, Li_2_N_2_, and LiN_3_, which had been synthesized successfully. Particularly, Lithium-to-nitrogen (Li:N) ratio equals one in Li_2_N_2_ which is neither Li-rich compound nor N-rich compound. In N-rich compound of LiN_3_, the polymerization of nitrogen was predicted above 375 GPa^12^. It is also expected that the polymerization of nitrogen forms in Li_2_N_2_ at high pressure. Li_2_N_2_ is obtained and crystallizes in Immm phase at high pressure and high temperature by decomposition of LiN_3_^13^. The nitrogen atoms form ${{\rm{N}}}_{2}^{2-}$ ions with the N=N double bonds in the orthogonal phase which is the first alkali diazenide. The compound containing the N=N double bonds is proposed as the precursor of the polymeric nitrogen, which may facilitate the formation of polymeric nitrogen under pressure. Also, it is possible to enhance the stability by introducing the metal element. Theoretically, Li_2_N_2_ consisting ${{\rm{N}}}_{2}^{2-}$ ion is proved to be one of the stable stoichiometries in Li-N system under the pressure range from 0 to 100 GPa^7,8^. Moreover, to the best of our knowledge, the polymerization of Li_2_N_2_ is not reported yet. Therefore, in this work, we systematically illustrate the phase transitions and bond features of Li_2_N_2_ at 0–300 GPa. Our results show that the polymerization of nitrogen in Li_2_N_2_ will occur at the pressure higher than 242 GPa.

## Methods

The crystal structures are the key to analyzing the properties of materials at high pressures. Here, the evolutionary algorithm (USPEX)^14,15^ combined with the first-principles calculations are adopted to search the structures of Li_2_N_2_. The fix-composition simulations are performed in the pressure range from 0 to 300 GPa. In the structural searches, the Perdew-Burke-Ernzerhof (PBE) exchange correlation functional^16^ and projector augmented wave (PAW) method^17^ are used as implemented in VASP code^18^. For Li and N atoms, 1*s*^2^2*s*^1^ and 2*s*^2^2*p*^3^ are treated as the valence electrons, respectively. After the structural searches are completed, the structures with the lowest enthalpies are re-relaxed with higher accuracy by employing the hard PAW pseudopotential of N atoms. The convergence tests are described in Fig. S1. The energy cutoff is set to be 1000 eV, and the k-points with 2π × 0.025 Å^−1^ resolution is automatically generated by Monkhorst-Pack scheme, at which the total energies are well converged. The relaxation is stopped until the force and the energy are converged to 1.0 × 10^−3^ eV/Å and 1.0 × 10^−6^ eV, respectively. To check the dynamically stability of the structures we obtained, the phonon calculation is carried out by Quantum ESPRESSO code^19^ based on density functional perturbation theory^20^.

## Results

**Figure 1:**
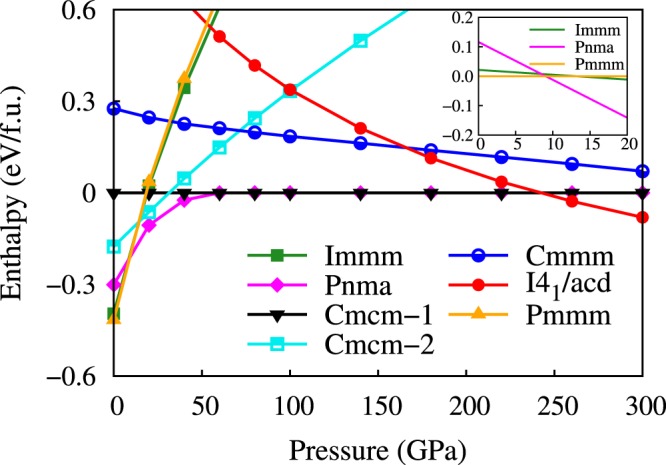
The enthalpy-pressure curve of the candidate structures refered to Cmcm-1 structure. There are two structures with the same symmetry Cmcm among all of the structures. In order to distinguish them, they are denoted as Cmcm-1 and Cmcm-2. The inset shows the enthalpies for Immm and Pnma structures relative to Pmmm structure at small pressure range.

**Figure 2:**
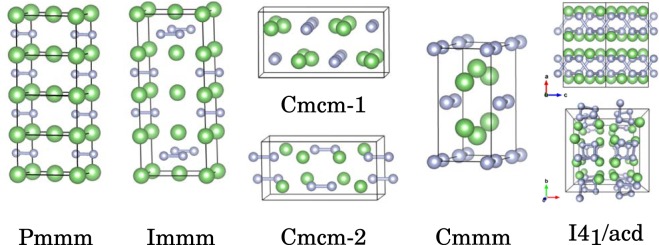
Crystal structures of Li_2_N_2_. In order to compare with Immm structure, the 1 × 1 × 4 supercell is used for Pmmm structure. The I4_1_/acd structure is viewed along *b* axis (1 × 1 × 2 supercell). Perspective view of the I4_1_/acd structure is also shown, looking down the nitrogen chains along *c* axis. The green and grey spheres represent Li and N atoms, respectively.

**Figure 3:**
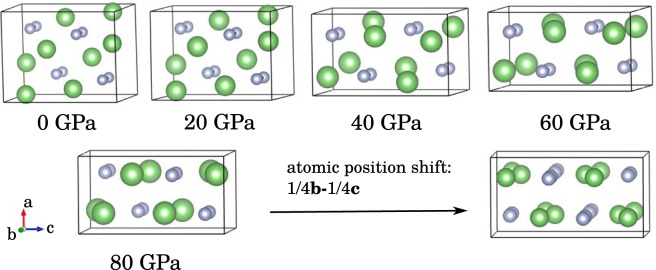
The evolutionary of Pnma structure at high pressures. When all of the atoms move along the direction 1/4**b**-1/4**c**, the equivalent unit cell of Pnma structure is obtained. It is easier to see the similarity between this Pnma structure and the Cmcm-1 structure shown in Fig. 2.

The structure searches are performed at 0, 5, 50, 100, 150, 200, and 300 GPa. The calculated enthalpies of candidate structures are shown in Fig. 1. At ambient pressure, the Pmmm structure of Li_2_N_2_ is the most stable phase, which is the same as that of Na_2_N_2_^21^. In our calculations, the energy difference between Pmmm and Immm is very small (2.8 meV/Li_2_N_2_) at 9 GPa, at which Immm structure was synthesized experimentally^13^. By considering the temperature effects in the experiment, Immm may become more favorable than Pmmm phase. As shown in Fig. 2, the difference of the two structures is the arrangement of N atoms in the frame formed by Li atoms. Pnma structure is the favorable phase above 9 GPa, and it transforms to Cmcm-1 structure with almost the same energy as Pnma above 60 GPa. In Pnma structure, the Li atoms are compressed into the same plane with the nitrogen atoms as increasing pressure, and the Pnma structure becomes Cmcm-1 structure finally (see Fig. 3). The phase transition order Pmmm → Immm → Pnma → Cmcm-1 agrees with the previous theoretical result at the pressure range of 0–100 GPa^8^, where the polymerization of nitrogen is not observed. In the following, we will discuss the behaviors of Li_2_N_2_ under the pressure higher than 100 GPa.

Our results show that Cmcm-1 structure transforms into the tetragonal phase I4_1_/acd at 242 GPa. Interestingly, the polymerization of nitrogen is observed in the I4_1_/acd phase, which contains the infinite N-chains with spiral structure (see Fig. 2). The polymerization pressure of 242 GPa in Li_2_N_2_ is much lower than that of LiN_3_ (375 GPa)^12^. At 300 GPa, the lattice parameters of I4_1_/acd structure are *a* = *b* = 6.16 Å and *c* = 3.10 Å with Li atoms at Wyckoff positions 16 *f* (0.39865, 0.64865, 0.125) and N atoms at Wyckoff positions 16 *f* (0.16073, 0.41073, 0.125). Besides, the results of the standard N pseudopotential are compared with that of the hard N pseudopotential. As shown in Figs 1, S2 and S3, the same order of phase transitions is obtained in the calculations by using both the standard N pseudopotential and the hard N pseudopotential. The pressure of Cmcm-1 → I4_1_/acd transition is 237 GPa in the calculations with the standard N pseudopotential, which is lower than the transition pressure obtained with the hard N pseudopotential. The small difference has marginal effect on the description of the phase transitions. Additionally, we consider the decomposition formula of Li_2_N_2_ → 2Li + N_2_, Li_2_N_2_ → 2/3LiN_3_ + 4/3Li, and Li_2_N_2_ → 2/3Li_3_N + 2/3N_2_, in which Fm $\bar{3}$ m, I $\bar{4}$ 3d, Aba2, and Cmca structures for Li^22,23^, P4_1_2_1_2, I2_1_3, Pba2, and I $\bar{4}$ 3m structures for N_2_^3,4^, C2/m and P6/m structures for LiN_3_^12^, P6/mmm, P6_3_/mmc, and Fm $\bar{3}$ m structures for Li_3_N^7,8^, are adopted. As shown in Fig. S4, the decomposition enthalpy is always higher than the lowest enthalpy curve, indicating that the Li_2_N_2_ is stable against decomposition.

**Figure 4:**
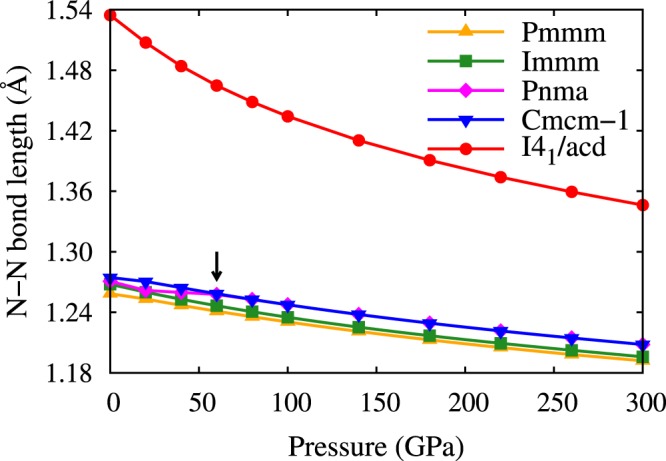
The variation of N-N bond length with pressure.

**Figure 5:**
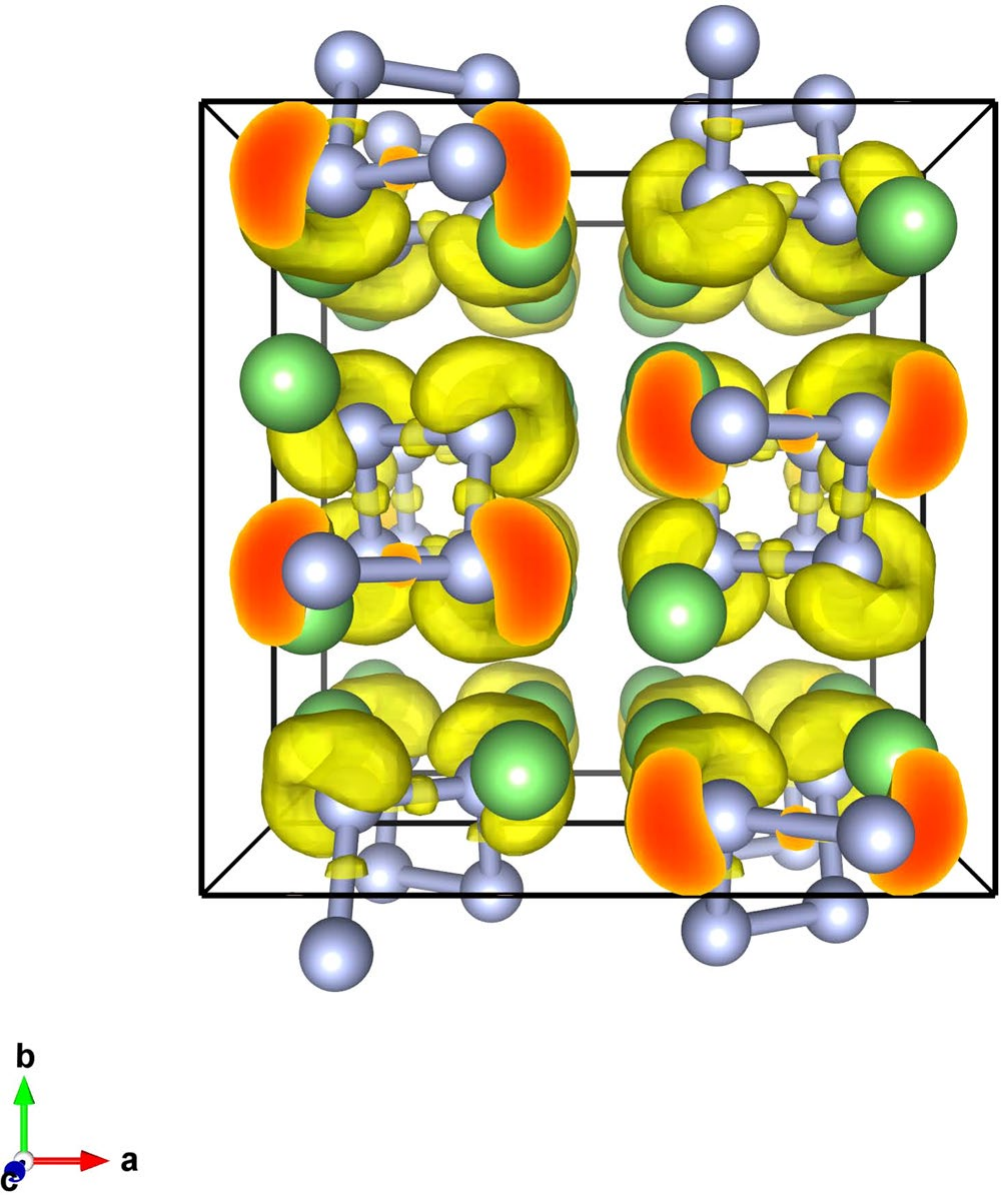
ELF plots of I4_1_/acd structure at 300 GPa. The isosurface value is set as 0.8.

The dependence of bond length on pressure is shown in Fig. 4. At ambient pressure, for Pmmm, Immm, Pnma, Cmcm-1, the N-N bond lengths are 1.258 Å, 1.268 Å, 1.271 Å, 1.274 Å, respectively, which are comparable to the N=N bond length (1.25 Å), indicating the existence of the N=N double bond in these phases. With increasing pressure, the N-N bond length is decreased sluggishly. Specially, the N-N bond length of Pnma is smaller than that of Cmcm-1 below 40 GPa, while above 60 GPa, the bond length of the two structures becomes the same, as indicated by an arrow in Fig. 4. This implies that the Pnma phase transforms to Cmcm-1 with increasing pressure, as shown in Fig. 3. In the I4_1_/acd structure, the nitrogen atoms form infinite spiral chains, and the bond length is 1.535 Å at 0 GPa which is bigger than the length of single bond (1.45 Å). The bond length decreases with increasing pressure. At 300 GPa, the N-N bond length becomes 1.346 Å, and the bond order^24^ is calculated to be 1.17, which is slightly larger than 1.0, confirming the single bonding nature. All of the N-N bond length has the same value in the spiral nitrogen chain, implying that the nitrogen atoms are equivalent. Furthermore, the equal bonding nature of all N atoms in a nitrogen chain is confirmed by the electron localization function (ELF) as shown in Fig. 5. Each N atom in the chains exhibits two N-N σ bonds and two lone pairs.

**Figure 6:**
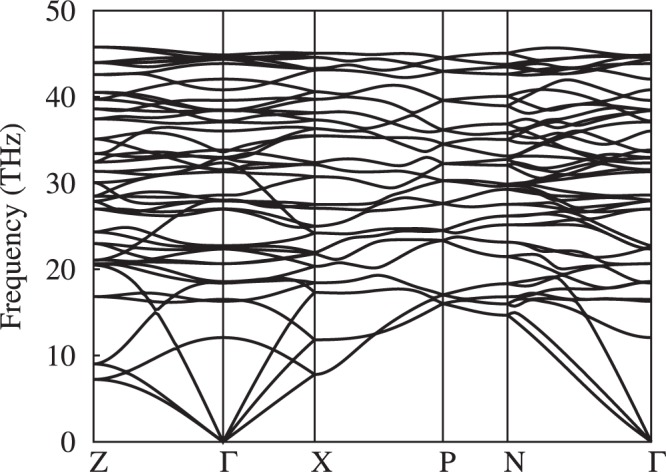
The phonon spectra of I4_1_/acd structure at 300 GPa.

**Figure 7:**
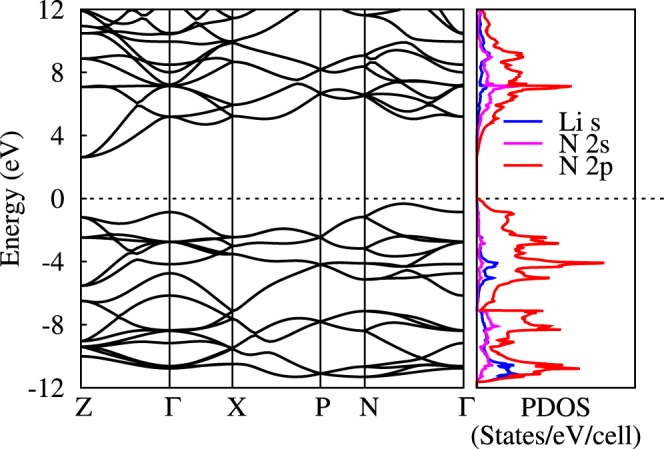
The electronic structure of I4_1_/acd phase at 300 GPa. The dotted line is the Fermi level.

The phase I4_1_/acd with polymeric chains is observed for the first time in Li_2_N_2_. The dynamical stability of I4_1_/acd structure is examined by phonon spectra using density functional perturbation theory. As shown in Fig. 6, there is no imaginary frequency in the whole Brillouin zone at 300 GPa, indicating the dynamical stability of I4_1_/acd structure. Since the primitive cell of I4_1_/acd has 16 atoms, the phonon dispersion curves consist of 3 acoustic branches and 45 optic branches. At Γ point, the frequencies of optic modes are 12 to 45 THz. When the pressure is decreased, the imaginary frequencies appear below 40 GPa, as shown in Fig. S5, which indicates that the structure is dynamically unstable at ambient pressure. Moreover, by using the substrate or nanostructured confinement^25,26^, the new polymeric phase may be stable at ambient condition. The calculated electronic band structure and density of states (DOS) for I4_1_/acd at 300 GPa are shown in Fig. 7, indicating that the I4_1_/acd is an insulator with the band gap of 2.7 eV at 300 GPa. The conduction band minimum locates at Z, while the valence band maximum appears along N-T. Therefore, the tetragonal phase with I4_1_/acd symmetry is descried as an indirect insulator. The electrons near Fermi level is mainly contributed by N 2*p* orbital and the contribution from Li is away from the Fermi level, indicating that Li_2_N_2_ is indeed a nitrogen dominated materials. The Bader charge analysis^27^ reveals that the charge transfer from Li atom to N atom is 0.8 *e*, suggesting the Li-N ionic bonding characteristic.

## Summary

In summary, the crystal structures of Li_2_N_2_ under high pressure are predicted by using evolutionary algorithm. Interestingly, the tetragonal phase I4_1_/acd featuring the polymeric nitrogen chains is identified for the first time in Li_2_N_2_. The transformation from ${{\rm{N}}}_{2}^{2-}$ ions to N chains is driven by pressure, implying that the N-N bonding pattern evolves from the double bond to the single bond. The infinite nitrogen chain is stabilized by introducing metal element, which transfers electrons to the nitrogen atoms. Also, it provides a suitable starting material for pursuit of nitrogen chain synthesis. This newly predicted tetragonal phase is energetically favorable above 242 GPa, which adds the understanding to the energetic nitrogen compounds under pressure.

## Electronic supplementary material

Supplementary information
