# Supplementary material for: The polymerization of nitrogen in Li2N2 at high pressures
Source: Sci Rep. 2018 Sep 3;8:13144. doi: 10.1038/s41598-018-31355-z (PMC6120935; doi:10.1038/s41598-018-31355-z)
Supplement: Supplementary file 1 — Supplementary information [file 41598_2018_31355_MOESM1_ESM.docx]

**The polymerization of nitrogen in Li_2_N_2_ at high pressures**

Jie Zhang^1^, Xianlong Wang^1,2^, Kaishuai Yang^1,2^, Ya Cheng^1,2^, Zhi Zeng^1,2,3*^

^1^Key Laboratory of Materials Physics, Institute of Solid State Physics, Chinese Academy of Sciences, Hefei 230031, P. R. China

^2^University of Science and Technology of China, Hefei 230026, China

^3^Beijing Computational Science Research Center, Beijing 100084, China

*E-mail: [zzeng@theory.issp.ac.cn](mailto:zzeng@theory.issp.ac.cn)

**1. The convergence tests with Li_sv and N_h pseudopotential**


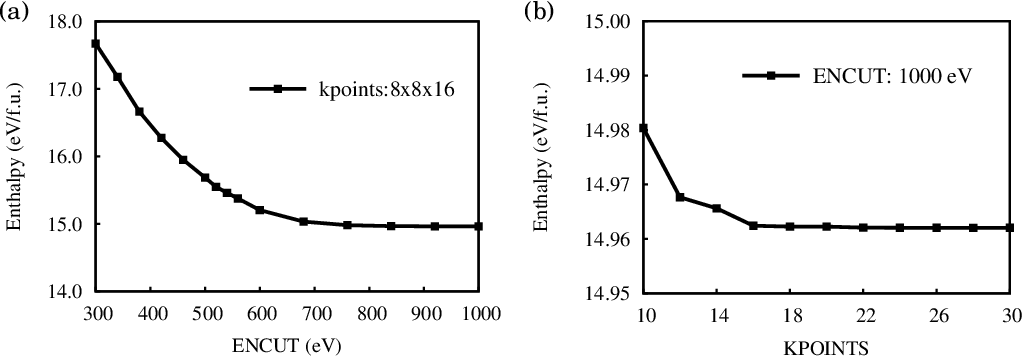


Fig. S1 The convergence tests of I4_1_/acd at 300 GPa with the hard N pseudopotential. (a) The convergence test for energy cutoff. (b) The convergence test for k-point grids. The KPONITS axis in (b) is determined by 2π/(*k* resolution).

**2. The calculated results with Li_sv and the standard N pseudopotential**

In the calculations, the energy cutoff and the k-points are chosen according to the convergence studies. Here, the results of convergence tests with I4_1_/acd structure are shown in Fig. S2. The energy cutoff with 520 eV and the k-point with 2π×0.03 Å^-1^ resolution are chosen, which ensures that the enthalpy is well converged. The enthalpy-pressure curve of the seven structures is plotted in Fig. S3. The tetragonal structure with I4_1_/acd symmetry is favored above 237 GPa.


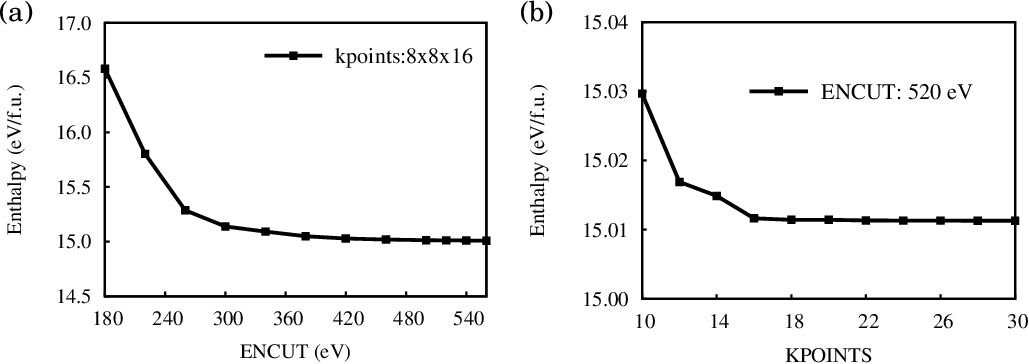


Fig. S2 The convergence tests of I4_1_/acd at 300 GPa with the standard N pseudopotential. (a) The convergence test for energy cutoff. (b) The convergence test for k-point grids. The KPONITS axis in (b) is determined by 2π/(*k* resolution).


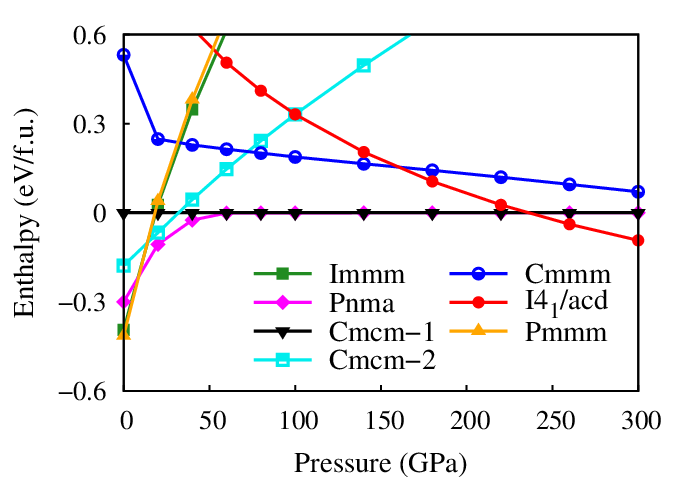


Fig. S3 The enthalpy-pressure curve of the candidate structures with the standard N pseudopotential. The Cmcm-1 structure is used as a reference. There are two structures with the same symmetry Cmcm among all of the structures. In order to distinguish them, they are denoted as Cmcm-1 and Cmcm-2.

**3. The decomposition enthalpies of Li_2_N_2_**


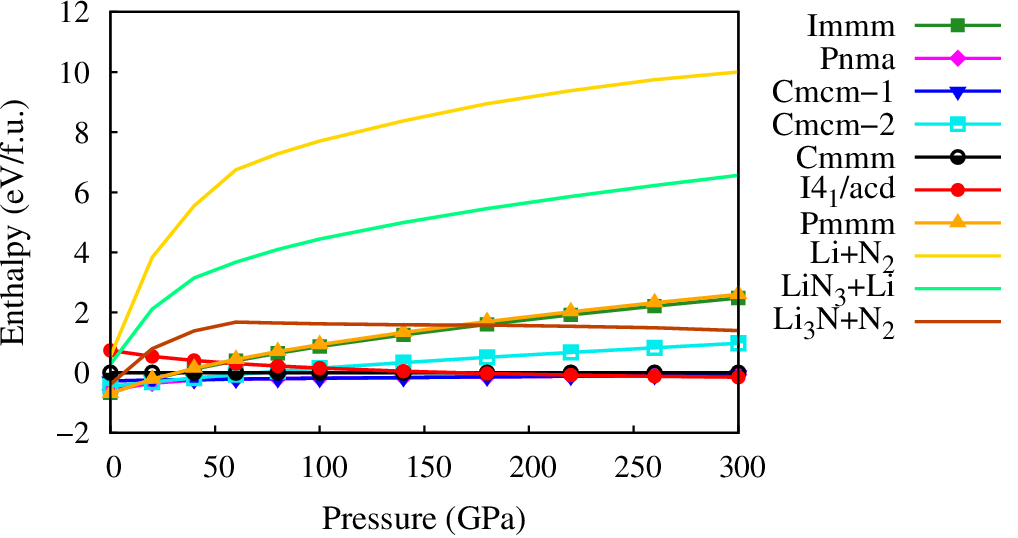


Fig. S4 The enthalpy-pressure curve of the candidate structures and the decomposition enthalpies referred to Cmcm-1 structure.

**4. The phonon spectra of I4_1_/acd atructure**

The phonon spectra are calculated by using a supercell approach as implemented in Phonopy code ^[^^[[1]](#endnote-1)]^.


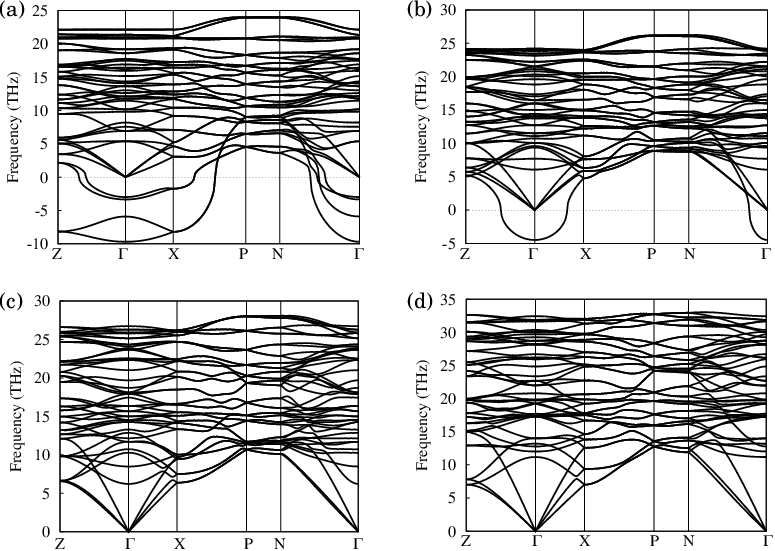


Fig. S5 The phonon spectra of I4_1_/acd structure at several different pressures. (a) 0 GPa, (b) 20 GPa, (c) 40 GPa, (d) 100 GPa.

1. [] Togo, A. & Tanaka, I. First principles phonon calculations in materials science. Scr. Mater., 108, 1-5 (2015). [↑](#endnote-ref-1)
